# Frequency domain measurements of melt pool recoil force using modal analysis

**DOI:** 10.1038/s41598-021-90423-z

**Published:** 2021-05-26

**Authors:** Tristan Cullom, Cody Lough, Nicholas Altese, Douglas Bristow, Robert Landers, Ben Brown, Troy Hartwig, Andrew Barnard, Jason Blough, Kevin Johnson, Edward Kinzel

**Affiliations:** 1 Department of Mechanical and Aerospace Engineering, Missouri University of Science and Technology, Rolla, MO 65409 USA; 2 Kansas City National Security Campus, Kansas City, MO 64147 USA; 3 Department of Mechanical Engineering-Engineering Mechanics, Michigan Technological University, Houghton, MI 49931 USA; 4 Department of Aerospace and Mechanical Engineering, University of Notre Dame, Notre Dame, IN 46556 USA

**Keywords:** Engineering, Mechanical engineering, Characterization and analytical techniques

## Abstract

Recoil pressure is a critical factor affecting the melt pool dynamics during Laser Powder Bed Fusion (LPBF) processes. Recoil pressure depresses the melt pool. When the recoil pressure is low, thermal conduction and capillary forces may be inadequate to provide proper fusion between layers. However, excessive recoil pressure can produce a keyhole inside the melt pool, which is associated with gas porosity. Direct recoil pressure measurements are challenging because it is localized over an area proportionate to the laser spot size producing a force in the mN range. This paper reports a vibration-based approach to quantify the recoil force exerted on a part in a commercial LPBF machine. The measured recoil force is consistent with estimates from high speed synchrotron imaging of entrained particles, and the results show that the recoil force scales with applied laser power and is inversely related to the laser scan speed. These results facilitate further studies of melt pool dynamics and have the potential to aid process development for new materials.

## Introduction

Understanding the complex melt pool dynamics in laser powder bed fusion (LPBF) processes is critical to maintaining quality of printed parts. Tight quality control is necessary as parts created by LPBF are used in demanding biomedical, aerospace, and defense applications. In the LPBF process a focused laser spot follows through a computer determined path over a thin powder layer. The powder is heated by the absorbed laser irradiation to the point that it melts and fuses with the underlying part. Variations in the melt pool dynamics lead to changes in microstructure, notably porosity. Specifically, insufficient laser fluence produces a lack of fusion between layers while excessive power leads to gas entrapment as the melt pool solidifies. The influence of the recoil pressure can be seen with the different melt pool modes. If the recoil pressure is too low, heat transfer in the melt pool operates in the conduction mode^1^, potentially leading to poor fusion between the molten material and the previous layer and result in brittle parts. Conversely, if the recoil pressure is too high, convection in the melt pool is the dominant heat transfer mode, depressing the melt pool into multiple previous layers and can potentially create keyhole porosity due to increased laser absorptivity and making the melt pool less stable^2,3^. This melt pool mode is referred to as the keyhole mode^4^. Since part quality is primarily driven by these defects, it is important to understand recoil pressure to operate the LPBF process between the conduction and keyhole modes, thus, minimizing the potential for these defects.

For most commercial processes, evaporation occurs at the melt pool surface, which produces a localized recoil pressure. This pressure depresses the melt pool^5^ which drives the melt pool deeper and enhances the heat transfer allowing for adequate layer-to-layer fusion.

The dynamics of the melt pool are driven by Marangoni^6–8^, capillary^9,10^ and buoyancy^11^ forces in addition to the recoil pressure. These forces all significantly influence the shape of the melt pool^7,8^ as well as its stability^6^ and overall fluid transport. However, the recoil force magnitude is significantly greater than the other forces acting on the melt pool once it starts to vaporize. For example, using the definitions of these forces described in Ref. ^12^ and typical processing parameters for 304L stainless steel^13^, the recoil force is at least an order of magnitude larger than the net Marangoni force, and more than eight orders of magnitude larger than the capillary and buoyancy forces.

The effects of the recoil pressure in depressing the melt pool has been studied numerically^7,14,15^. While modeling recoil pressure is computationally expensive, an estimate for the recoil pressure can be made using the Clausius-Clapeyron model and via the simulation results in Khairallah et al.^16^. The recoil pressure was estimated to be 86 kPa for a 316L stainless steel simulation with a laser power of 200 W and a scan speed of 1.5 m/s. Simulations have also shown that as material is vaporized, changes in the thermal and fluid transport within the melt pool lead to surface defects known as humps^10,17^. This is an effect of the backflow generated by the recoil pressure as the exposed area of the melt pool is displaced away from the center. In addition, the recoil pressure driven melt pool depression has been experimentally shown to be correlated with the formation of spatter^18–25^, as well as influence the magnitude of the oscillations that occur within the melt pool^26^.

Recently, there have been a few attempts to measure recoil pressure using particle tracking techniques. This requires in-situ high-speed imaging of the melt pool. Zhao et al. used a custom built 2D setup illuminated with synchrotron radiation to estimate an average pressure above the melt pool of 60 kPa for Ti-6Al-4 V powder melted with a laser power of 210 W and a scan speed 0.5 m/s^27^. Yin et al. calculated a vapor pressure of 49 kPa for Inconel powder processed with a laser power of 1150 W, a scan speed of 1 m/s, and a spot size of 159 µm by observing spatter tracks using high-speed visible camera imaging^28^. These studies pose instrumentation challenges and require the assumption that the particles are moving parallel to the imaging plane. In addition, significant complications arise with the presence of the gas that flows over the build plate (i.e., shielding gas) to create an inert atmosphere as it substantially modifies particle velocities. Shielding gas was present during the experiment in the Inconel study^28^ to prevent melt pool oxidation while the Ti-6Al-4V study was performed in vacuum^27^.

A possible alternative to measuring the laser spatter trajectories is to directly measure the reaction force generated by the recoil pressure. Both experimental and numerical studies give an expectation for the recoil pressure on the order of 50–90 kPa. This is equivalent to a recoil force acting on the melt pool in the sub mN range. Measuring this force in the time domain is difficult given the noise inherent in LPBF environments (e.g., shielding gas, chiller). However, if the experiments can be conducted in the frequency domain, spectral filtering techniques can be employed to significantly improve the Signal to Noise Ratio (SNR). This paper presents a study in a commercial LPBF system using an accelerometer to measure part vibration and quantify recoil force in the frequency domain. Modal analysis is used in this study by exciting resonant parts with a laser to calculate the recoil force. This approach is used to measure the recoil force for various ranges of process parameters to evaluate their relationship with recoil pressure. Finally, the dependence of the microstructure and melt-pool depth on recoil pressure for typical LPBF of SS304L parts is presented.

## Experimental approach

The experiments in this paper are conducted using a commercial LPBF machine (AM250, Renishaw). This machine stabilizes the melt pool using an Acousto-Optic Modulator (AOM) to pulse the laser. During processing, the laser is pulsed for duration *τ*_*pulse*_ ~ 75 µs while being held stationary at a point. At the conclusion of the pulse, galvo scanners move the beam along the scan path by a point distance, *PD*^29,30^. The point distance and pulse duration can both be adjusted to provide an estimation of the scanning velocity, *V* = *PD*/*τ*_*pulse*_. Adjusting the pulse length corresponds to specifying the pulse repetition frequency (PRF) of the laser modulation, 1/*τ*_*pulse*_, and can be adjusted from 1 to 25 kHz with *τ*_*pulse*_ adjustable in 10 µs increments.

**Figure 1:**
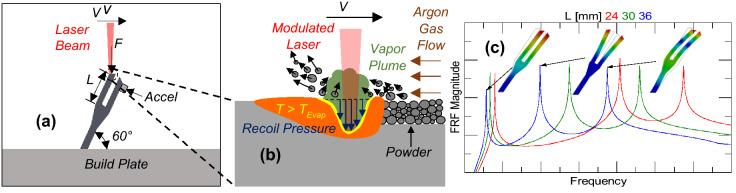
Illustrations of **(a)** laser excitation of tuning fork with accelerometer (Accel) mounted on tuning fork prong, **(b)** laser interaction with powder on top of tuning fork during laser excitation, and **(c)** example FRFs and mode shapes.

Figure 1 shows a part being excited by the laser. In the LPBF process, the laser energy is absorbed by the layer of unfused powder on top of the part. The powder is melted and then vaporized. The metallic vapor exerts a recoil pressure on the melt pool, deforming it and producing a net force normal to the surface. In addition to the vaporized metal, particles can be entrained by the local pressure field and ejected away from the melt pool. This is depicted in Fig. 1b.

**Figure 2:**
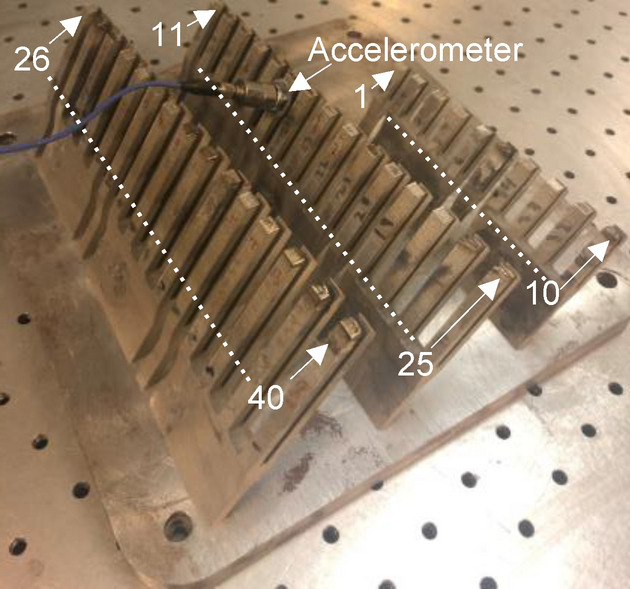
Tuning fork build with arrows indicating beginning and ending tuning forks for each row, where the tuning fork numbers linearly increment. The tuning forks that were used in experiments are tabulated in Table 1 in “Methods”.

The experiments conducted in this paper use 304L stainless steel powder. Tuning forks are printed directly on the build plate. After printing, the unfused powder is removed from the chamber and an accelerometer is fixed to the tuning forks as illustrated in Fig. 1a and shown in Fig. 2. Experiments show that the powder ordinarily surrounding the part increases structural damping by a factor of 14, which would significantly lower the sensitivity of the experiment. After fixing the accelerometer to the tuning fork with superglue, a new 50 µm thick layer of powder is placed on the top surface of the part. The force generated by the laser interaction at the melt pool produces a strong acceleration response when the part is forced at resonance. This requires both the force and the accelerometer to be coupled to a common resonant mode. To accomplish this, the tuning forks are printed at 60° relative to horizontal so the laser would excite the bending modes and the parts could be printed without support structures. The force/acceleration coupling of the tuning forks was simulated using ANSYS as shown Fig. 1c and is quantified by the Frequency Response Function (FRF)

1 $$ FRF\left( f \right) = {{a\left( f \right)} \mathord{\left/ {\vphantom {{a\left( f \right)} {F\left( f \right)}}} \right. \kern-\nulldelimiterspace} {F\left( f \right)}} $$

where *f* is the frequency, *a* is the part acceleration, and *F* is the force applied to the part. The tuning fork modes in Fig. 1c were using ANSYS. The mass of the accelerometer was not included in the ANSYS simulations.

Experimentally, the force acting on the part consists of all of the melt pool forces; including capillary (i.e., surface tension), thermo-capillary (i.e., Marangoni), and recoil^5,31,32^. However, as discussed earlier, the recoil force magnitude is expected to dominate those of the other forces. The FRF of each tuning fork was measured experimentally with a modal impact test by striking each prong with an impact hammer and measuring the corresponding acceleration. The impact hammer strikes the same surface that is irradiated by the laser and as normal to the surface as possible (see Fig. 1a). This test occurs with the build plate inside the LPBF chamber. Powder is than added to the top surface of part, the chamber is evacuated and back filled with Argon, and the acceleration measured while the part is scanned by the laser with the accelerometer in the same position. After the test, the FRF was measured again to ensure that any changes resulting from the laser interaction (e.g., additional mass fused to the tip of the tuning fork) are negligible.

Multiple tuning forks are fabricated in order to have resonant frequencies spanning a wide range of laser PRFs. Because the FRF of the individual parts are known (after modal impact hammer testing), the PRF used to excite an individual tuning fork is adjusted to match the tuning fork’s resonant frequency. Figure 2 shows a photograph of 40 tuning forks, 12 of which used in the experiments. During the experiment, the accelerometer remained in the same location during the laser excitation and the impact test afterwards. Since the location of the accelerometer did not change during the test, the relative effect of the mass loading is effectively cancelled out. The tuning forks are printed using a laser power *P* = 175 W, *V* = 0.8 m/s, exposure time of 75 µs, hatch spacing of 85 µm, and *PD* = 60 µm. The resonant frequencies of the 12 turning forks are given in Table 1 of the Methods section.

**Table 1:** Tuning forks used in experiments, resonant frequencies, and prong lengths.

Tuning fork	Resonant frequency (kHz)	*L* (mm)	Mode number
15	10.05	35	7
16	10.03	36	8
17	9.930	37	12
20	9.800	41	12
27	6.300	49	11
29	6.300	51	10
30	6.175	52	10
31	11.02	53	14
32	5.200	54	11
33	5.490	56	9
34	6.256	57	12
39	2.050	62	7

**Figure 3:**
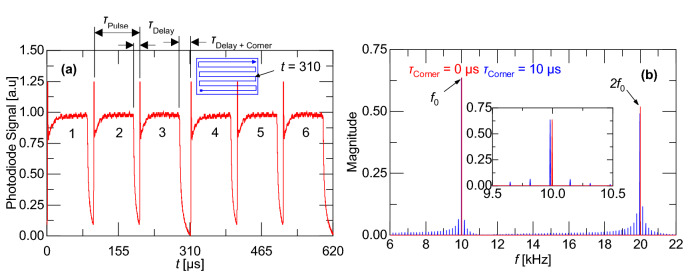
**(a)** Normalized experimental photodiode waveform with inset showing raster scan path for τ_Pulse_ = 100 µs and **(b)** corresponding signal in the frequency domain with and without cornering.

The laser power modulation in the AM250 is not an ideal rectangular wave. Figure 3a shows the response from a photodiode exposed to scattered laser radiation. The measured laser/AOM response has rise and fall times of ~ 10 µs. In the experiment shown in Fig. 3a, the laser is scanned over an alumina disk using *τ*_Pulse_ = 100 µs. Between pulses, there is a delay time of *τ*_Delay_ = 10 µs. The duty cycle is 1 – *τ*_Delay_/*τ*_Pulse_. The figure also shows that at *t* = 310 µs the effect of the laser as it reverses its travel as part of the raster scan process (scan path illustrated in the inset). This introduces a slightly longer delay (*τ*_Delay+Corner_ ~ 20 µs) before the next pulse. The variable *τ*_Delay_ is a function of the point distance but is constant for PD < 60 µm, while *τ*_Delay+Corner_ is a function of point distance and hatch spacing. Both of *τ*_Delay_ and *τ*_Delay+Corner_ are kept constant in this paper. The addition of the intermittent delay associated with cornering introduces a phase delay in the frequency domain. Figure 3b shows the Fourier transform of the measured laser/AOM response from a single line with and without cornering. The fundamental frequency without cornering occurs at *f*_0_ = 10 kHz with harmonics at integer multiples of the fundamental frequency (red curve). However, the Fourier transform of a waveform including raster scanning (i.e., with cornering) shows a shift in the fundamental frequency and a slight (0.5%) decrease in magnitude. This energy loss is shifted to side bands (blue curve).

A range of laser powers, pulse durations, and scan paths were recorded. As Fig. 3b shows, much of the laser energy is outside of the measurement range. Assuming that the recoil force has the same frequency content as the laser/AOM response, the measured recoil force at the fundamental frequency can be scaled by the ratio of the laser energy at the fundamental frequency to the total laser energy. This fraction varies with the duty cycle and the scaling is described in the Methods section. Further, for the experiment conducted in this study, to prevent aliasing of the PRF peak magnitude, the frequency resolution, *df,* is selected such that it is an integer multiple of the PRF, i.e., rem(PRF/*df*) = 0.

## Results and discussion

### Single line scan path without powder

**Figure 4:**
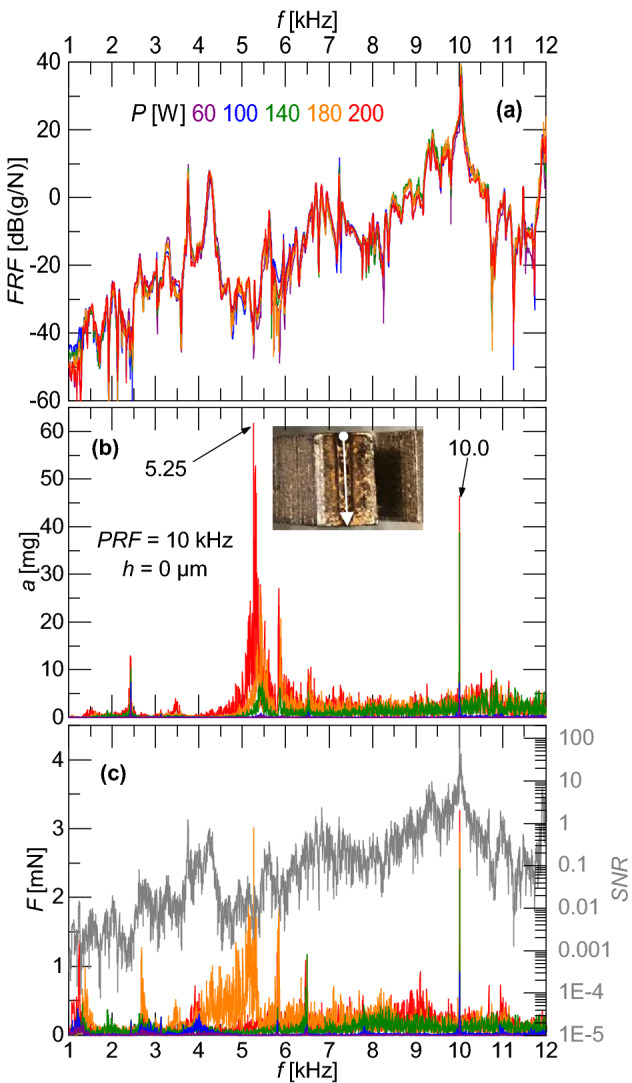
Results for Tuning Fork #16 **(a)** measured FRFs for various laser powers, **(b)** measured acceleration spectrum with inset showing single line scanning strategy schematic, and **(c)** calculated forcing spectrum with SNR spectrum (gray line). V = 10 mm/s.

The simplest case occurs when no powder, i.e. *h* = 0 µm, is added to the exposed surface of the tuning fork and the scan path consists of a single line to avoid cornering. A laser PRF of *f*_0_ = 10 kHz and a point distance of *PD* = 1 µm, corresponding to a scan speed of *V* = 10 mm/s, are used. This scan speed is well below the range typical for 304L stainless steel^13,33^; however, it was selected to gather sufficient data when processing a single line (i.e., the laser did not need to reverse its direction of travel ). Figure 4 shows experimental results for a Tuning Fork using various laser powers. Because of the atypically high linear energy density, the FRF was measured after each experiment to determine if the sample was physically modified (i.e., its frequency response changed). Figure 4a shows that the resonant frequency changed over a range of only 8.7 Hz during the five experiments. Further, the peak response at *f*_*r*_ = 10.03 kHz is approximately 18 dB larger than the next highest peak, demonstrating that most of the energy is contained at this frequency. The measured acceleration during laser excitation is shown in Fig. 4b. While there is energy at other frequencies, this shows a very sharp peak at the excitation frequency which matched the resonant frequency of the part. The value of the acceleration also scales with the laser power, whereas the energy at other frequencies does not. This is due to the fact that the laser is only providing energy at 10 kHz and the subsequent harmonics; therefore, the only portion of the acceleration that should be changing with laser power is at 10 kHz. Figure 4c shows the force response calculated by solving Eq. 1 for *F*(*f*) using the measured FRF and acceleration. An equivalent Noise Equivalent Force (NEF), the ratio of the measured accelerometer noise to the difference between the FRF signal and the measured FRF noise as described in the Methods section, is used to calculate an equivalent Signal to Noise Ratio (SNR). Figure 4c shows that this is much higher near the part resonance and supports the confidence of the magnitude of the force at the laser PRF (more than five orders of magnitude greater SNR at *f* = 10 kHz than *f* = 5.25 kHz).

**Figure 5:**
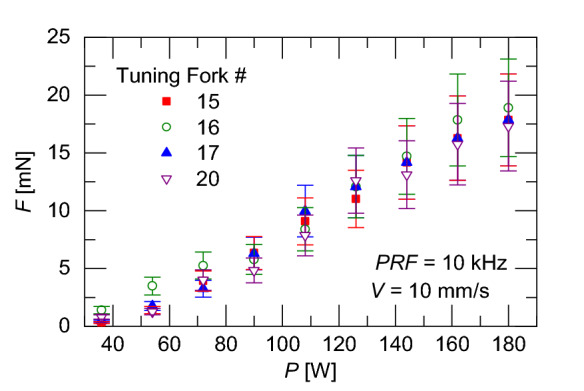
Recoil force versus applied laser power for single line scanning strategies using Tuning Forks #15, #16, #17, and #20.

To examine the part independence of this result, the experiment is repeated with different tuning forks using the same laser powers. The different tuning forks have slightly different FRFs with resonant frequencies listed in Table 1 in the “Methods” section. Figure 5 shows the total recoil force as a function of laser power after correcting for the fraction of the force at the fundamental PRF frequency. The corrective measure for the fraction of force at the PRF frequency is shown in the Methods section in Fig. 12. Figure 5 shows that the recoil force scales with the laser power with good agreement across multiple specimens. The increase in recoil force is due to the fact that as the laser power increases a greater amount of material will be vaporized.

### Rasters with powder

**Figure 6:**
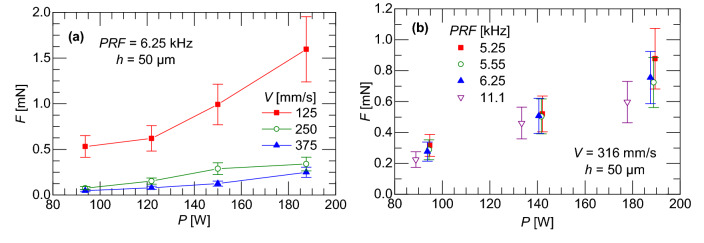
**(a)** Recoil force as function of laser power and scan speed for constant PRF and **(b)** recoil force as function of applied laser power for constant scan speed. Uncertainty is detailed in “Methods” section.

The slow scan speed and the lack of powder in Figs. 4 and 5 does not correspond to typical LPBF processing conditions. Figure 6 shows the recoil force for a range of characteristic process parameters for 304L stainless steel^34^. A powder was spread across the surface of the tuning forks using two 50 µm thick metal shims to create a uniform layer. The laser was rastered with scan speeds varied by changing the point distance. The higher scan speeds, compared to that used in the previous section, required rastering the laser; therefore, the recoil force was corrected using the measurement at the fundamental frequency to account for the delay time introduced by cornering. The force correction for the additional delay time is shown in the Methods section in Fig. 12. Figure 6a shows the variance in the measured recoil force with respect to scan speed and laser power when the PRF is maintained at 6.25 kHz. The magnitude of the force is inversely proportional to scan speed, which agrees with the results in Figs. 4 and 5 where an order of magnitude slower scan speed produced recoil forces that were an order of magnitude higher. It is significant to note that the recoil force does not depend appreciably on the PRF. This is illustrated in Fig. 6b where the scan speed is constant while the laser PRF and laser power are varied. These results demonstrate that when the PRF is varied but the scan speed remains constant, the material vaporization remains constant in the process parameter range considered in this study.

**Figure 7:**
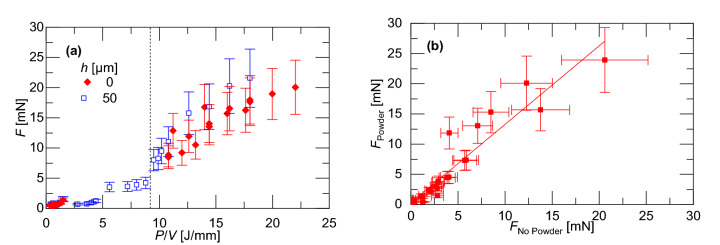
**(a)** Recoil force as function of linear energy density for both powder and no powder being and **(b)** recoil force magnitude comparison for both powder and no powder.

Figure 7 shows the data in Figs. 4, 5 and 6 replotted as a function of the linear energy data, *P*/*V*. Single line scans at slow scan speeds with powder are also included in the figure. There is minimal recoil force at very low linear energy densities (notably, the data between *P*/*V* = 2 and 5 J/mm is generated using a laser power of *P* = 40 W). However, lager linear energy densities, *P*/*V* > 9.2 J/mm, produce a significantly greater recoil force. This is consistent with models of the LPBF process in Trapp et al.^3^, predicting that the greater temperatures produced by higher linear energy densities increase the vaporization rate. The greater vaporization rate produces a higher recoil pressure, forming a cavity which traps more laser energy to further increase the absorptivity of the melt pool. This positive feedback on the recoil pressure leads to formation of keyholes. In general, the figure shows that the addition of powder increases the recoil force by a factor of 1.33 from the case without powder. This can be attributed to a greater absorptivity of the loose powder bed and the fact that isolated powder particles are more readily vaporized because of reduced thermal conductance to surrounding material.

### Measurements of melt pool after solidification

**Figure 8:**
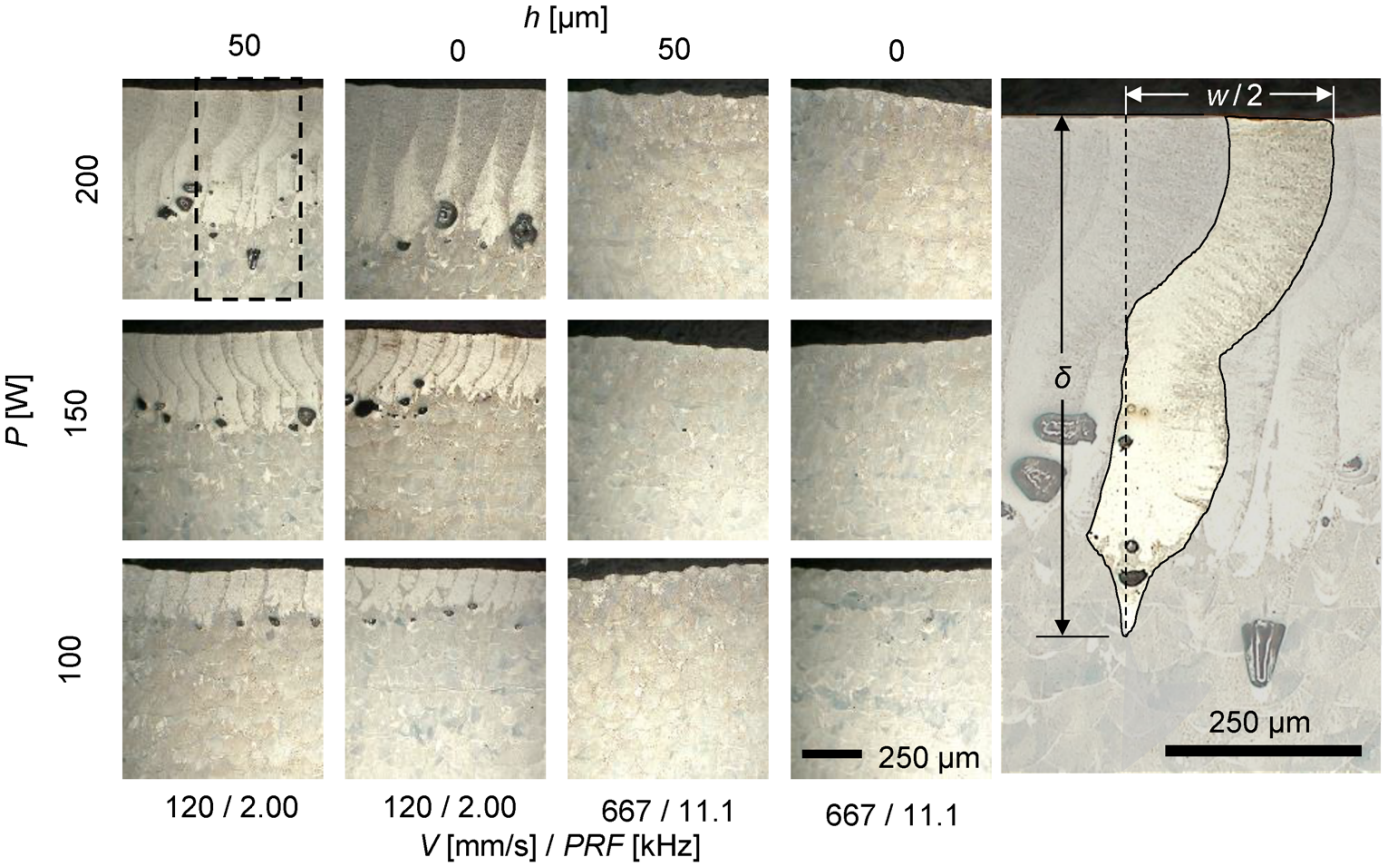
Sample micrographs for different laser powers, scan speeds, and PRFs with melt pool dimensions annotated in side picture that is annotated portion of melt pool indicated by dashed box.

**Figure 9:**
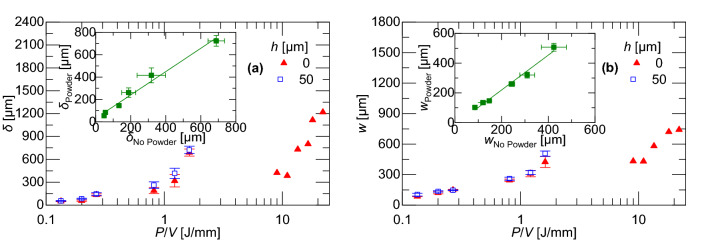
Melt pool **(a)** depth and **(b)** width as function of linear energy density for experiments with powder and no powder. Insets show melt pool dimensions for experiments with powder versus experiments without powder.

The recoil force significantly affects the melt pool morphology. Figure 8 shows melt pool metallographic micrographs for different process parameters obtained from an auxiliary set of experiments (different substrates but the same processing conditions as the samples in Figs. 2, 3, 4,5, 6 and 7, see “Methods” section). The melt pool depth, *δ*, and half-width, *w*/2, are defined in the close up of the metallograph for *P* = 200 W and *V* = 200 mm/s in Fig. 8. As expected, the melt pool width and depth increase with increasing laser powers and decrease with increasing scan speed. Most significantly, gas porosity can be seen with lower scan speeds. These are plotted as a function of linear energy density in Fig. 9. It is interesting to note that the melt pool dimensions produced in experiments with and without powder are linearly related. This is plotted in the insets of Fig. 9 where the melt pool depth for experiments with powder is 1.11 times the melt pool depth for experiments without powder, and the melt pool width for experiments with powder 1.12 times the melt pool width for experiments without powder. This agrees with the increased recoil forces measured for experiments with powder. The results indicate that increasing melt pool dimensions without keyholing or significant recoil pressure for experiments with powder can be attributed to increased absorptance of the powder bed, which leads to greater heating, and thus size, of the melt pool.

**Figure 10:**
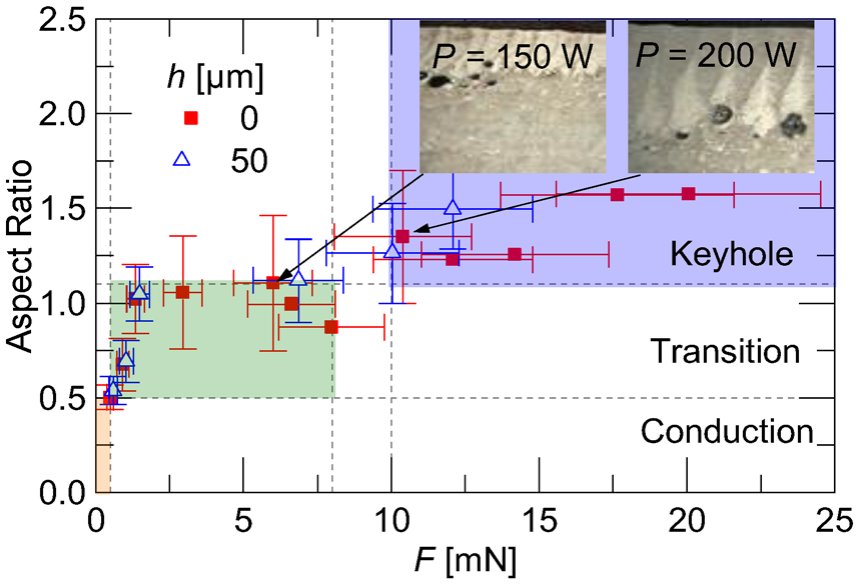
Melt pool aspect ratio as function of recoil force with horizontal lines indicating dominant melting modes and vertical lines representing recoil force magnitudes at melting mode boundaries with insets showing micrographs that were collected at *V* = 120 mm/s and *PRF* = 2 kHz.

The melt pool aspect ratio (AR), *δ*/*w*, has been shown to be correlated with different melting modes^1^. Specifically, Qi et al.^35^ gives ranges for the conduction, transition, and keyhole mode of *AR* ≤ 0.5, 0.5 < *AR* < 1.1, and 1.1 ≤ *AR*, respectively. Figure 10 shows the measured aspect ratio as a function of the measured recoil forces. Vertical dashed lines show the ranges of recoil forces producing each mode. Again, this agrees with the metallography in Fig. 8 (close-ups shown in the insets of Fig. 10) showing the gas porosity resulting for slow scan speeds and void size scaling with laser power.

None of the process parameters produced *AR* < 0.5, which corresponds to conduction mode. For process parameters with *AR* ~ 0.5, the recoil force was measured to be less than 0.5 mN. This appears to be a threshold between conduction and transition melting modes, and recoil forces between 0.5 and 8 mN produced aspect ratios in the range between 0.5 and 1.1, meaning these forces were in the transition region. Basing the definition of the keyhole mode as the melt pool ratio of > 1.1 from Qi et al.^35^, it was empirically found that this corresponds to a measured force of 10 mN. It is worth noting that with the results from Yin et al. ^26^, who estimated a pressure of 49 kPa over a laser spot size of 159 µm, corresponding to a recoil force of 0.97 mN in the transition region which agrees with our experimental findings. It is significant to note that the presence of powder during processing does not significantly appear to change these thresholds.

## Summary and conclusions

This paper demonstrated a method to measure the recoil force produced by the recoil pressure in LPBF using a vibration response approach. While subject to well bounded error, the results correlate well with microstructure analysis. The following conclusions can be drawn from the study:

- Recoil force is proportionate to the linear energy density past a threshold where the powder begins to be melted.
- The recoil force increases by 33% with the addition of a 50 µm layer of powder on the part surface compared to a bare surface.
- Melt pool depth and width scale with linear energy density for experiments both with and without powder.
- The process is hypothesized to operate in the conduction, transition, and keyholing modes for recoil force values less than 0.5 mN, between 0.5 and 8 mN, and greater than 10 mN, respectively.

## Methods

### Equipment

**Figure 11:**
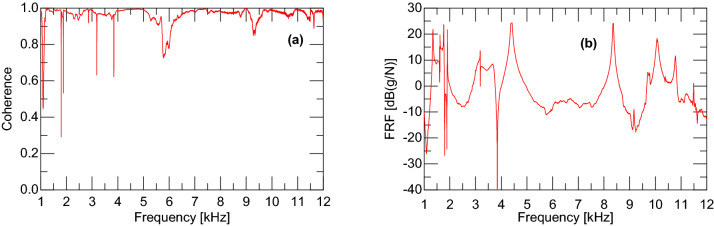
**(a)** Coherence spectrum and **(b)** FRF for tuning fork 15.

The accelerometer used in this study was model 352C34 from PCB Piezotronics. The bandwidth of the accelerometer was 0.005–12 kHz. The impact hammer used to measure the sample FRFs was model 086E80 from PCB Piezotronics. This impact hammer is rated to typically excite frequencies up to 12 kHz given a metal tip. The coherence spectrum to support this is shown in Fig. 11, which was the coherence plot for the response from Tuning Fork #15. The coherence response has notches which correlate to the anti-resonances in the FRF response. Aside from the anti-resonances, the magnitude of the coherence is above 0.95, indicating good correlation between the impact and the resulting acceleration.

The photodiode used to capture the waveforms was model PDA100A2 from Thorlabs. The bandwidth of the photodiode was 11 MHz.

### Tuning fork dimensions

The prong lengths and resonant frequencies of the samples used in the experiments are tabulated in Table 1.

### Materials

The material used in this study was 304L stainless steel purchased from LPW Technology.

### Recoil force magnitude correction

**Figure 12:**
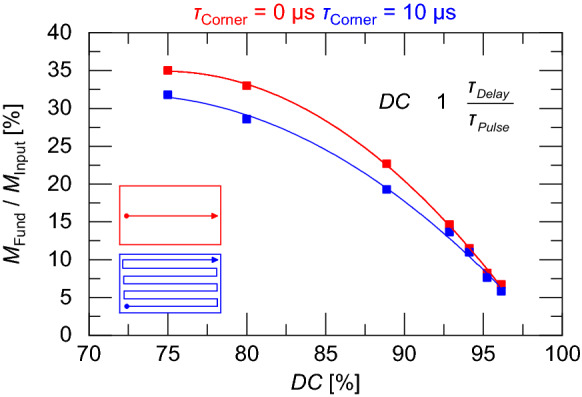
Percentage of energy located in fundamental frequency for various duty cycles and both straight (red) and raster (blue) paths.

For most of the samples in Table 1, the higher harmonics lie outside of the bandwidth of the accelerometer used in this study. The total energy can be inferred from the energy in the first harmonic assuming that the force response scales with the laser energy as measured by a photodiode response. This gives the fractional energy in the first harmonic a dependence on the duty cycle of the laser’s pulse train. The fractional energy was calculated by taking the ratio of the magnitude at the fundamental frequency to the rms magnitude of the input signal i.e. *M*_Fund_/*M*_Input_. Figure 12 shows the fraction of energy in the first harmonic relative to the total energy of the measured photodiode signal for various laser duty cycles.

Using the data in Fig. 12, the total estimated recoil force is,

2 $$ F = \frac{{a_{{f_{1} }} \left( f \right)}}{{FRF_{{f_{1} }} \left( f \right)}}\frac{{M_{Input} }}{{M_{Fund} }} $$

where *a* is the measured tuning fork acceleration magnitude during the laser excitation at the first harmonic, and *FRF* is the experimentally measured tuning fork FRF magnitude at the first harmonic.

### Uncertainty calculation

The uncertainty in the recoil force measurements was defined using the following propagation of uncertainty

3 $$ F_{Unc} \left( f \right) = \left| F \right|\sqrt {\left( {\frac{{E_{Accel} \left( f \right)}}{A\left( f \right)}} \right)^{2} + \left( {\frac{{E_{Force} \left( f \right)}}{F\left( f \right)}} \right)^{2} } $$

where *E*_*accel*_ and *E*_*Force*_ are the margins of error for a 95% confidence interval, defined below, for the measured FRFs and how the acceleration spectrums were processed. The margin of error is defined as

4 $$ E = Z_{\alpha /2} \frac{\sigma }{\sqrt N } = 1.96\frac{\sigma }{\sqrt N } $$

where *Z* is the normal distribution indexed at the confidence level of interest, *σ* is the standard deviation of the FRFs, and *N* is the number of FRFs taken.

### Auxiliary experimental procedure for melt pool dimensions

Rectangular prisms were printed of dimensions 6.35 × 3.85 × 5 mm. The process parameters used to print the specimens were *P* = 200 W, *V* = 0.8 m/s, exposure time of 75 µs, hatch spacing of 85 µm, and *PD* = 60 µm. After they were printed, their top surfaces were scanned with the same laser powers, scan speeds, and PRFs as the ones seen in Figs. 6, 7 and 8a. After scanning, the specimens were removed from the build plate, mounted using a Simplimet 1000, polished using an AutoMet 250 Grinder-Polisher, and then subsequently etched using a 40/60 nitric acid solution. The melt pool dimensions were measured using a Hirox KH-8700 digital microscope.
